# Game Changer: Exploring the Role of Board Games in the Lives of Autistic People

**DOI:** 10.1007/s10803-024-06408-0

**Published:** 2024-07-05

**Authors:** Liam Cross, Francesca Belshaw, Andrea Piovesan, Gray Atherton

**Affiliations:** 1 Department of Psychology, University of Plymouth, Plymouth, PL4 8AA UK; 2 Department of Psychology, Edge Hill University, Liverpool, L39 4QP UK

**Keywords:** Autism, Board games, Adults, Hobbies, Mental health, Wellbeing

## Abstract

This mixed methods paper reports findings from three studies examining the overlap between autism and hobbyist board gaming. The first was a quantitative survey of over 1600 board gamers, showing that autistic individuals are overrepresented in this hobby compared to the general population and that autistic traits measured by the AQ are significantly elevated amongst board gamers. Study 1 also assessed gamers’ motivations and preferences and reported key differences as well as similarities between autistic and non-autistic gamers. The second was a qualitative study that reported the results of 13 interviews with autistic individuals who are hobbyist board gamers. Using Interpretive Phenomenological Analysis (IPA), four key themes were uncovered, including a preference for systemising, escapism and passions, the social lubrication effect of games and difficulties with deception. In the third, 28 autistic individuals were introduced to board games in groups of 5–10 over an afternoon. Subsequent focus groups were then analysed using IPA. This analysis uncovered themes around how board games are challenging but encouraged growth and how they were an alternative vehicle for forging social relationships. Through this paper, we discuss how and why board games may be a popular hobby amongst the autistic population, and its potential utility for improving autistic wellbeing.

## Introduction

Autism spectrum condition (ASC) is a neurodevelopmental condition affecting an estimated 1% of the population globally (Kogan et al., 2018). A recent systematic review by Zeidan et al. (2022) estimated that the median prevalence of autism worldwide is 100/10,000 (1% prevalence), with a median male-to-female ratio of 4.2 to 1 and co-occurring intellectual disability at around 33%. Great strides have been made to improve awareness and acceptance of autism, including reconceptualising autism as a condition with considerable accompanying strengths (Cope & Remington, 2022). Nevertheless, there is still a need to understand the strengths and challenges inherent to the autistic experience to improve the quality of life throughout the lifespan, as research suggests that autistic adults do not experience the same gains as neurotypicals when moving through adulthood (Atherton et al., 2021).

### Flow Theory, Monotropism and Passions

Around 75–90% of autistic people, compared to an estimated 30% of neurotypicals (Klin et al., 2007), report having strong interests in domains where they develop expertise and high levels of engagement (Caldwell-Harris & Jordan, 2014). These are traditionally referred to as restricted interests in the DSM V, though here we use the term passions as this is a strengths-based term (Bailey, 2023; Barton & Hamilton, 2012). Two theories have been used to understand the hyper-focus often seen in autistic people when engaged with their passions. Csikszentmihalyi and Csikszentmihalyi, (1990) described flow as a psychological state in which one achieves a high level of enjoyment of a task to the point of experiencing optimal happiness where nothing else seems to matter. An increasingly popular model for autism that can describe this atypical focus of attention is the interest model, also known as monotropism (Murray et al., 2005). In a monotropic flow state, autistic people can gain predictability, achievement, and optimal happiness by being ‘pulled in’ by their passions (Milton, 2017). Some have argued that monotropic attention may be advantageous to autistic people when it is channelled to provide educational and social advantages, such as developing expertise and demonstrating enthusiasm (Wood, 2021).

Though autism can be understood as a condition with considerable accompanying strengths, research suggests that there are still struggles autistic people face in everyday life (Graham Holmes et al., 2020). To compensate for these, many autistic people become adept at masking or camouflaging, which means hiding one’s autistic traits in order to fit into a neurotypical world (Hull et al., 2017). Masking is related to poor mental health outcomes (Bradley et al., 2021), including an increased risk for negative self-appraisal (Cage & Troxell-Whitman, 2019) and engaging in self-harm (Mournet et al., 2023). One method for living authentically is for autistic people to be open about their areas of expertise or interests, which is positively linked to their quality of life (Grove et al., 2018). Passions engage and motivate autistic individuals and often reduce stress and anxiety (Attwood, 2003). For example, Winter-Messiers (2007) found that when autistic students were involved in activities related to their passions, they reported higher self-esteem, felt more confident and displayed more enthusiasm and positive emotions. Autistic individuals often report the need to express their desires and interests to allow them to feel comfortable in social situations and their environments (Späth & Jongsma, 2020). In this sense, it is vital to encourage the passions of autistic people and create opportunities for these passions to be explored in social spaces.

### Autism and Board Games

Board gaming is a pastime that may be particularly well suited to autistic monotropic engagement, as it requires sustained attention and a transfer of established skills to new domains (Gobet et al., 2004). As autism is a particularly heterogeneous condition, a characteristic which extends to the diversity of passions in those on the spectrum (Nowell et al., 2021), the variety of board game options may be particularly well suited to this population (Brown & MacCallum-Stewart, 2020). The wide array of board games on offer means autistic people can find a game that suits their unique interests. For instance, research on the most common passions of autistic people includes animals and transport (Cho et al., 2017). These are also common themes of board games (Cross et al., 2023), which may mean that autistic people could find game themes that allow them to engage with their areas of expertise, which has been shown to benefit autistic well-being (Harrop et al., 2019).

Autistic individuals often struggle to form close relationships and friendships, with research suggesting they are more likely to feel lonely and isolated (Mazurek, 2014; Umagami et al., 2022). Board games may be a vital hobby to improve these outcomes. Rogerson et al. (2016), for instance, interviewed eleven board gamers who stressed the importance of board games sociality, highlighting how spending time with like-minded people was a crucial aspect of play.

Though there is very little academic work in this area (Atherton & Cross, 2021), there is a great deal of anecdotal evidence that modern board games may be a popular hobby for those on the spectrum. Multiple magazine articles and blog posts discuss the link between the two (Russell, 2023), and there are myriad examples of overlap between autism and modern board gaming in popular media (Arndt, 2023).

### Modern Board Gaming

Modern board gaming is a fast-growing hobby, and its community is evolving rapidly, having achieved unparalleled popularity and commercial success in the last twenty years. In 2016, The Guardian reported on ‘The Rise and Rise of Tabletop Gaming,’ citing related social and design factors underpinning this surge in interest. While board games may have previously been synonymous with childhood, consumer demographics of modern board games are decidedly adult (Woods, 2012). They include young professionals, including couples, who prefer to play games with friends rather than go out to pubs or clubs. They often overlap with ‘geek culture,’ or individuals who are also interested in computers, video games, science fiction and comics (Woo, 2012). With the general acceptance of games in the broader culture, including those accessible on mobile platforms like smartphones and browsers, gamification within Western culture provides fertile ground for the continued proliferation of board games. Market research predicts a $4 billion growth in the global board games market from 2020, reaching $30 billion by 2026 (Arizton Advisory Intelligence, 2020). Millions attend conventions like GenCon, Spiel and the UK Games Expo annually. With the rise of game cafes, the growing acceptance and self-identification of ‘geek’ or ‘nerd culture’ (Kinney, 1993; Woo, 2012), and the pandemic spurring at-home forms of entertainment (Coward-Gibbs, 2022), board gaming is gaining popularity and visibility.

### Purpose of the Current Studies

To date, there is a limited amount of research exploring the impact board gaming might have on the social lives of autistic individuals. This work, therefore, aims to address this gap in three ways: (1) By exploring the representation of autistic people in the hobby, (2) By understanding what it is autistic board gamers get out of the hobby, and (3) By introducing autistic people not already involved in the hobby to it, to understand if and how it could be beneficial to them. This paper reports three studies examining the relationship between autism and board games to better understand the potential benefits of board gaming for autistic individuals. Study 1 assessed the prevalence of autistic individuals and those with higher autistic traits in this hobby, as well as gamer preferences and motivations. Study 2 explored the lived experience of 13 autistic gamers through interviews. Study 3 introduced groups of autistic people to board games and then examined their utility through focus groups.

## Study 1

This work set out to investigate the prevalence of autism amongst board game hobbyists and evaluate whether this is indeed a leisure activity that is common in the autistic population (as anecdotal evidence suggests). A large dataset which surveyed hobbyist board gamers (Cross et al., 2023) was utilised to establish the prevalence of mental health conditions and other demographics in this population. Preferences for game styles, themes and mechanics, and gamers’ motivations for playing were also explored. These findings offer clinicians and educators interested in utilising board games in their work valuable data about the games that autistic individuals most and least enjoy. This dataset is open access on the open science framework (https://osf.io/vygd3/?view_only=d1d52d8e0fca4e98be9c5c4dd54e846b).

### Methods

This study utilised a survey design administered on Qualtrics. A target sample of 1500 board gamers was solicited, and data collection was left open for two months. Participants were recruited from special interest groups for board gamers on social media, and further invites were sent out to gamers from industry mailing lists. This call was explicitly addressed to those already involved in the hobby. However, as we wanted to assess the rate of autism naturally present amongst this population, the call did not mention autism, and autistic participants were not directly recruited. Each participant was given a digital copy of a board game in return for participation. Respondents were surveyed on their demographics and preferences in the hobby. All measures and response formats are reported briefly below, and a full copy of all questions and answers can be found in the supplementary materials. More details on the design and data can be found at Cross et al. (2023).

RQ1: Is autism more or less prevalent among board gamers than in the general population?

RQ2: Do the motivations and preferences for board gaming differ between autistic and non-autistic players?

#### Demographics

Respondents first reported gender, biological sex assigned at birth, age, ethnicity, nationality, educational level and diagnosed mental health conditions. These were answered using drop-down sections using the standard Qualtrics pre-sets. Those who indicated they had a diagnosis of ASC were asked to specify if they received that diagnosis from a medical professional and at what age they were diagnosed. All participants also completed the Autism Quotient (AQ), a commonly used 50-item measure of autistic trait levels (Baron-Cohen et al., 2001).

#### Gamers’ Experiences

Respondents were then asked to report their general experiences with playing board games. This included their level of familiarity with games (newbie/novice, casual, midcore/core or hardcore/expert), the number of hours played on average per month (< 1, 1–4, 5–9, 10–19, 20–29, 30–39, 40 +), and their preferred platform (online, in-person or both equally). Then, participants were asked to rate their enjoyment of several gaming elements such as preferred player count; game length, pieces (i.e., cards, dice, etc.), style (competitive, cooperative, etc.), classification (Euro, Ameri, Hybrid), and type (gateway, party, heavy, etc.), on a slider scale from ‘not at all’ to ‘very much’. Next, respondents were asked to rate their preferences on how much they enjoyed 28 board game mechanics (an industry/hobby-specific term referring to the rules and actions that keep the game moving towards a victory, i.e., dice rolling, worker placement, area control, player elimination, etc.) again from ‘not at all’ to ‘very much.’ Following this, respondents rated their enjoyment of the 14 most popular board gaming themes (as indicated by BoardGameGeek.com, i.e., war, crime, farming) on the same slider scale. Respondents then indicated (via similar sliding scales) how important (not important—very important) several aspects were when choosing a game (i.e., theme, components, mechanics, etc.) and what motivates them to play a game (competition, socialising, escapism, etc.). Respondents then indicated how important gaming was for their social life and how important it was to feel like a part of the board gaming community. All slider scales generated a number from 0 to 100 (which was not visible to participants) and were presented with the anchor point positioned in the middle of the scale, which needed to be moved before the page could progress. Definitions of all relevant terms and example games were provided alongside each question. For a full copy of the measures, please see the supplementary materials.

#### Participants

A total of 1603 individuals completed the questionnaire, specifically 1242 males and 361 females aged between 18 and 73 years old (mean age = 32.38; SD = 9.21), with ethnicities of White (60.6%), Asian (34.1%), Black (1.4%), Hispanic (1.1%), Middle Eastern (0.6%), and Other (2.2%). across 63 different countries, with a concentration of participants from the US (11.2%), UK (27.4%), France (18.3%) and China (25.5%). Participants showed a high level of education (37.7% reported being university graduates, and 20.7% held a postgraduate degree). Edge Hill University’s ethics committee granted full ethical approval, and all participants gave informed consent.

### Results & Discussion

#### Mental Health and Neurodevelopmental Conditions

Alpha levels of Mann–Whitney U tests reported below were not corrected for multiple tests as these were exploratory analyses. Maintaining a 0.05 alpha level will provide further insights to explore in future studies. As shown by Table 1, most participants (72.9%) indicated having no mental health or neurodevelopmental condition. In contrast, just over one-fourth of participants reported having at least one medically diagnosed mental health/neurodevelopmental condition. Of the respondents, 4.7% of the sample reported having autism, with Clopper-Pearson’s exact method suggesting 3.70 and 5.83% as the lower and upper limits for the population proportion with 95% level of confidence. This statistic is much higher than the estimated global prevalence rate of 1%, according to 99 estimates from a systematic review of 71 papers (Zeidan et al., 2022). The prevalence of individuals with autism in our sample is also higher compared to studies that looked at adults exclusively and reported a prevalence of 1.1% (95% CI: 0.3–1.9%; Brugha et al., 2011). Additionally, research suggests that autism rates are highest in Western countries (for instance, the prevalence in Asia is 0.36%) (Qiu et al., 2020). As such, our data suggests that autism rates among board gamers are significantly higher than is typically found in the general adult population worldwide. In a similar vein, given the complexity of many board games and the cognitive skill level required to play them, it is unlikely that individuals with intellectual disability (where comorbidity with autism is an estimated 25% (Idring et al., 2015) to 33% (Zeidan et al., 2022) would be represented in this online sample.

**Table 1:** Mental Health Conditions Frequency in the Sample

Condition	Frequency (%)
None	1157 (72.9)
Autism	75 (4.7)
Dyslexia	68 (4.2)
ADHD	62 (4.1)
Depression	210 (13.2)
Anxiety	196 (12.2)
Other	29 (1.8)
Not specified	18 (0.4)

Some participants indicated more than one condition

Our sample showed a typical prevalence of individuals with ADHD. A total of 4.1% (2.98–4.93 Clopper-Pearson’s 95% confidence limits) of participants reported a diagnosis of ADHD, which is in line with reported prevalence in adult general population of 2.5% (95% CI 2.1 – 3.1) to 5.2% reported by others (95% CI 4.6–5.8) (Fayyad et al., 2007; Simon et al., 2009). As past literature has found significant comorbidity between autism and ADHD in the general population (around 50%; Rong et al., 2021), we checked this comorbidity in our sample. Among individuals with a diagnosis of autism, 9.3% of individuals reported also having ADHD. This was higher than the frequency of ADHD in TD individuals (2.7%) and BAP individuals (5.8%). However, 9.3% comorbidity is significantly lower than what has been found in past studies looking at autism in the general population (around 50%; Rong et al., 2021), which may suggest that autistic board gamers are a unique group (discussed further in the discussion).

Similarly, participants with dyslexia were 4.2% (3.25–5.28 Clopper-Pearson’s 95% confidence limits), a similar prevalence rate to what is estimated in the general population.

Shaywitz and Shaywitz (2005) suggested that the prevalence of dyslexia is between 5 and 17% of school-age children in the United States, while, although the prevalence in adulthood is less studied, it is thought to be around 4% (DSM-V, as cited by Soriano-Ferrer & Martínez, 2017).

The most common mood disorder was depression, with 13.2% (11.49–14.85 Clopper-Pearson’s 95% confidence limits) of the sample reporting having received a diagnosis. This is in line with the estimates suggested by Lim et al. (2018) of 12.9%, and higher compared to the 8.1% estimates of depression prevalence among adults (20^+^yo) in the USA between 2013 and 2016 (Brody et al., 2018). Anxiety was the second most common condition reported by 12.2% of participants (10.60–13.87 Clopper-Pearson’s 95% confidence limits). This was higher than what was reported by previous research that suggested that the current global prevalence of anxiety disorders adjusted for methodological differences was 7.3% (4.8–10.9%) and ranged from 5.3% (3.5–8.1%) in African cultures to 10.4% (7.0–15.5%) in Euro/Anglo cultures (Baxter et al., 2013). The prevalence of anxiety in our sample was also higher than that recorded in adults exclusively, which has been estimated to be 3.8–10.4% in Euro/Anglo cultures and 2.8% in Asian cultures (Remes et al., 2016).

#### Autism Quotient

Research suggests that many adults may have autism, but due to age and other variables, a formal diagnosis is often missed (Lai & Baron-Cohen, 2015). Therefore, we also explored the level of autistic traits self-reported by our sample. We were interested in exploring the relationship between board gaming and individuals with subclinical autistic traits, known as the Broad Autism Phenotype (BAP). The BAP refers to elevated but subclinical levels of autistic traits commonly possessed by close relatives of people with a clinical diagnosis of autism (Losh et al., 2011).

Participants’ mean AQ dichotomous score was 21.36 (SD: 7.09; median: 22, range: 1–45). A Wilcoxon signed-rank test indicated that the AQ dichotomous score of our sample was significantly higher than 19.38 (*Z* = 848643, *p* < 0.001), which is the mean AQ dichotomous score of 450,394 people reported by Ruzich et al. (2015). The number of respondents who scored above the clinical cut-off of 32 was then calculated, indicating individuals who would be highly likely to have or receive a clinical diagnosis of autism (Woodbury-Smith et al., 2005). 107 participants (6.7%) had a dichotomous score equal to or higher than this cut-off score. If used as a proxy for the likelihood of an autism diagnosis, this suggests that autism is more than five times higher in this sample than the global prevalence rate of 1%. Next, we assessed the proportion of people who display elevated but not clinical levels of autistic traits, scoring in the Broader Autism Phenotype range of above 26 using the original cutoff scores for the BAP (Baron-Cohen et al., 2001). A total of 467 participants (29.1%) were included in the BAP range. The frequency of individuals scoring in the BAP range was far greater than the scores found in students in science fields (15.4%) and non-science fields (8.3%) (Baron-Cohen et al., 2001). To compare gamer motivations and preferences we split our sample into two groups: an ASC group (160 participants, 10% of our sample), which included everybody who reported having a diagnosis of autism and those who had a dichotomous score equal to or above the clinical cut-off point of 32 on the AQ, and a TD group (1443 participants), which included the remaining participants. A Kruskal–Wallis test (*X*^2^(2) = 926.8, *p* < 0.001) and pairwise comparisons (all *p*s < 0.001) confirmed that AQ total scores were significantly higher in people with a diagnosis of autism (mean: 144.2; SD: 8.8; median: 143.0; range: 131–176) compared to BAP individuals (mean: 129.7; SD: 4.7; median: 129; range: 117–144), which, in turn, had higher scores compared to neurotypicals (mean: 110.4; SD: 11.7; median: 112; range: 71–132).

#### Summary

The only neurodevelopmental condition which appeared elevated in our sample compared to the general population estimate was autism with 4.7% of board gamers in our sample reported having a clinical diagnosis of autism compared to the general population estimate of 1% (Zeidan et al., 2022). Equally, analyses showed that the average AQ score of this population was higher than the general population, with 6.7% of the sample scoring above the clinical cut-off and 29.1% in the BAP range. Those who reported having a medical diagnosis of autism, combined with those that scored above the clinical cut-off point for the AQ, equalled 10% of the total sample. These findings show that, as hypothesised, the proportion of autistic individuals and individuals with elevated levels of autistic traits are over-represented amongst board gamers compared to the general population.

#### Gamers’ Experience

A significant Pearson’s Chi-Square (Table 2) suggested that participants in the ASC group (those with a clinical diagnosis of autism or those scoring above the clinical cut-off in the AQ) had more board game experience than the non-ASC group. In total, 62.8% of autistic gamers consider themselves midcore or hardcore players, while only 50.0% of the non-ASC group considered themselves as such. A significant Pearson’s Chi Square also indicated that the ASC group (53.1%) preferred to play online over the Non-ASC group (40.4%). There was no significant difference in the number of hours played between the two groups.

**Table 2:** Frequency (and percentage) of participants’ experience as board gamers divided by TDs and ASCs

		Non ASC	ASC	Chi square
Player experience	Newbie/Novice	128 (8.9)	21 (13.1)	11.58**
	Casual	409 (28.3)	59 (36.9)	
	Midcore/core	581 (40.3)	57 (35.6)	
	Hardcore/Expert	325 (22.5)	23 (14.4)	
Hours played	< 1 h	108 (7.5)	15 (9.4)	6.94
	1-4 h	200 (13.9)	30 (18.8)	
	5-9 h	287 (19.9)	36 (22.5)	
	10-19 h	384 (26.6)	38 (23.8)	
	20-29 h	246 (17.0)	25 (15.6)	
	30-39 h	96 (6.7)	6 (3.8)	
	40^+^hr	122 (8.5)	10 (6.3)	
Preferred platform	Online	201 (13.9)	32 (20.0)	10.00**
	In person	860 (59.6)	75 (46.9)	
	Both equally	382 (26.5)	53 (33.1)	

Pearson’s Chi square tests are included

*p < .05; **p < .01; ***p < .001

#### Game Preferences

Mann–Whitney U tests (Table 3) indicated that the non-ASC group preferred to play with 3 or more players, while this was rated lower for those in the ASC group. Meanwhile, those in the ASC group liked to play alone more than those in the non-ASC group. The ASC group also reported a preference for cooperative games over the various forms of competitive games, a preference not seen in the non-ASC group. Similarly, those in the ASC group reported a greater dislike for lighter social/party games compared to those in the non-ASC group.

**Table 3:** Participants’ median ratings for game details divided by TDs and ASCs

		Non ASC^	ASC^	Mann–Whitney U
Number of players	3–4 players	87	80	94,626.5***
	2 players	75	75	111,861.5
	5 + players	69	57.5	97,240**
	1 player	51	61.5	130,592**
Game length	30-60 min:	80	76	106,470.5
	1-2 h:	78	76.5	112,478
	< 30 min:	71	71	116,629.5
	2 + hr:	53	58	107,982
Game elements	Boards	80	72	98,822.5**
	Cards	79	75.5	103,713*
	Dice	67	63	108,180
	Hybrid (with app or web)	56	51	107,296
Game style	Competitive (all vs all)	81	73	98,094**
	Cooperative	79	78	113,934
	Cooperative (with traitor)	71	62	101,192*
	Team	69	64.5	102,271*
	Competitive (1 vs all)	61	59.5	103,239.5*
Game classification	European	81	76	102,720.5*
	Hybrid	77	74.5	106,062.5
	American	68	66.5	107,461
Game types	Heavy	75	74.5	113,098.5
	Gateway	71	70	104,971.5^+^
	Party	68	63	100,189**
	Abstract	65	64	110,531.5

Mann–Whitney U Tests are Included

^+^*p* = .059; * *p* < .05; **p < .01; ***p < .001

^Minimum and maximum ratings were always 0 and 100

#### Mechanics

**Fig. 1:**
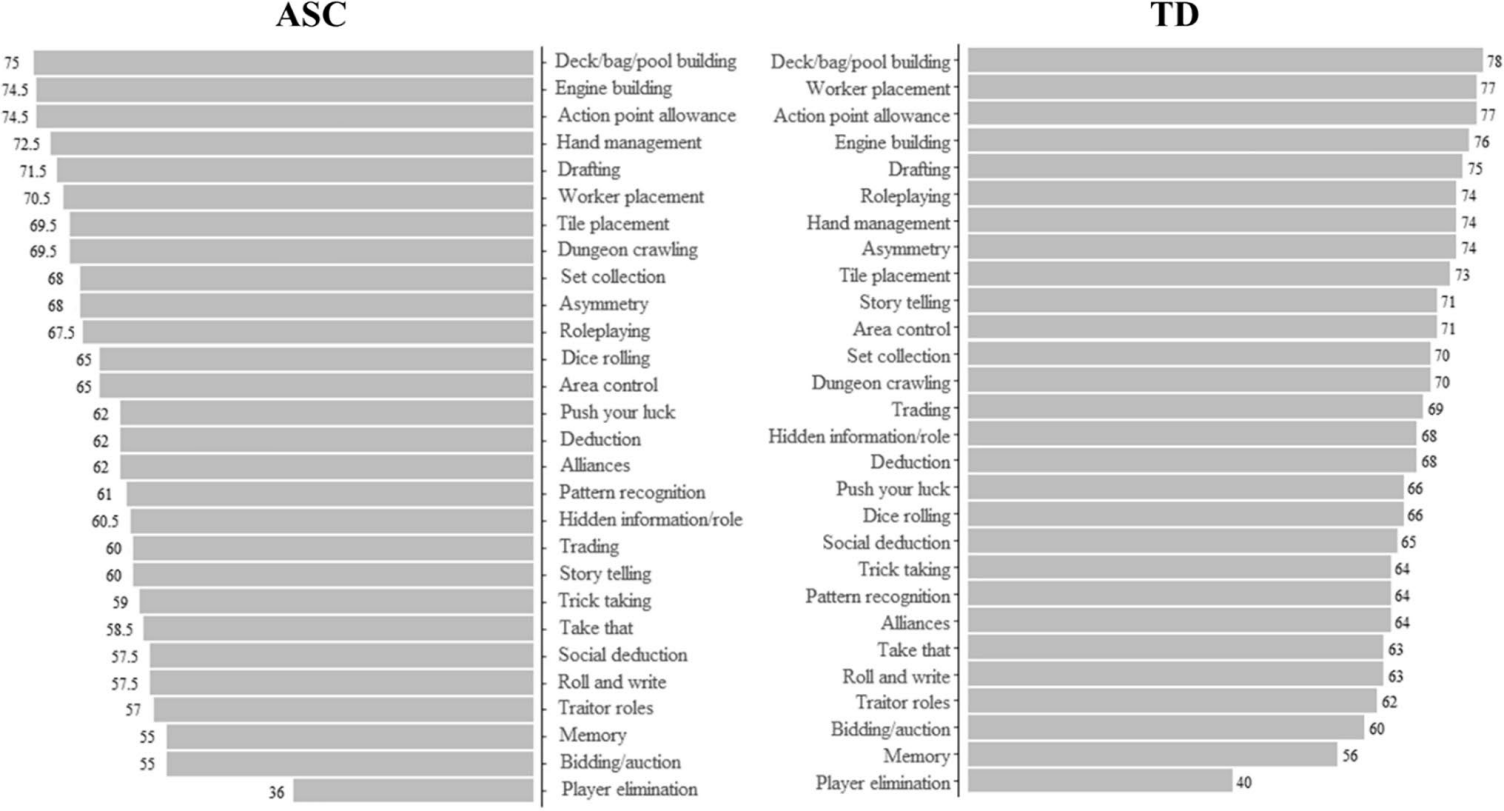
Median Ratings for Game Mechanics in Order for Each Group

Although Mann–Whitney U tests (Table 4 and Fig. 1) indicated that those in the ASC group consistently gave lower ratings than those in the non-ASC group, the rating order was similar between the non-ASC and ASC groups, with few notable exceptions. Autistic players ordered engine building, hand management, tile placement, set collection and dungeon crawling mechanics as more preferable than Non-ASC players. Those in the ASC group also showed a reduced preference for certain social elements, including storytelling, trading, social deduction, deduction, and hidden information games.

**Table 4:** Median ratings for board game mechanics divided by TDs and ASCs

Mechanics	Non ASC^	TD^	Mann–Whitney U test
Deck/bag/pool building	75	78	113,656
Action point allowance	74.5	77	105,230.5
Engine building	74.5	76	112,815.5
Hand management	72.5	74	111,554
Drafting	71.5	75	104,158.5*
Worker placement	70.5	77	97,686**
Tile placement	69.5	73	106,023
Dungeon crawling	69.5	70	117,073.5
Asymmetry	68	74	106,363
Set collection	68	70	107,387.5
Roleplaying	67.5	74	102,538.5*
Area control	65	71	103,393*
Dice rolling	65	66	113,974
Deduction	62	68	96,451***
Push your luck	62	66	104,111*
Alliances	62	64	104,838^+^
Pattern recognition	61	64	112,013.5
Hidden information/role	60.5	68	96,671.5***
Story telling	60	71	91,079***
Trading	60	69	89,483***
Trick taking	59	64	100,141.5**
Take that	58.5	63	104,577.5^++^
Roll and write	57.5	63	102,889.5*
Social deduction	57.5	65	94,884.5***
Traitor roles	57	62	101,176*
Bidding/auction	55	60	101,432.5*
Memory	55	56	110,363
Player elimination	36	40	109,268.5

Mann–Whitney U Tests are Included

^+^*p* = .056; ^++^*p* = .050; *p < .05; **p < .01; ***p < .001

^Minimum and maximum ratings were always 0 and 100

#### Themes

**Fig. 2:**
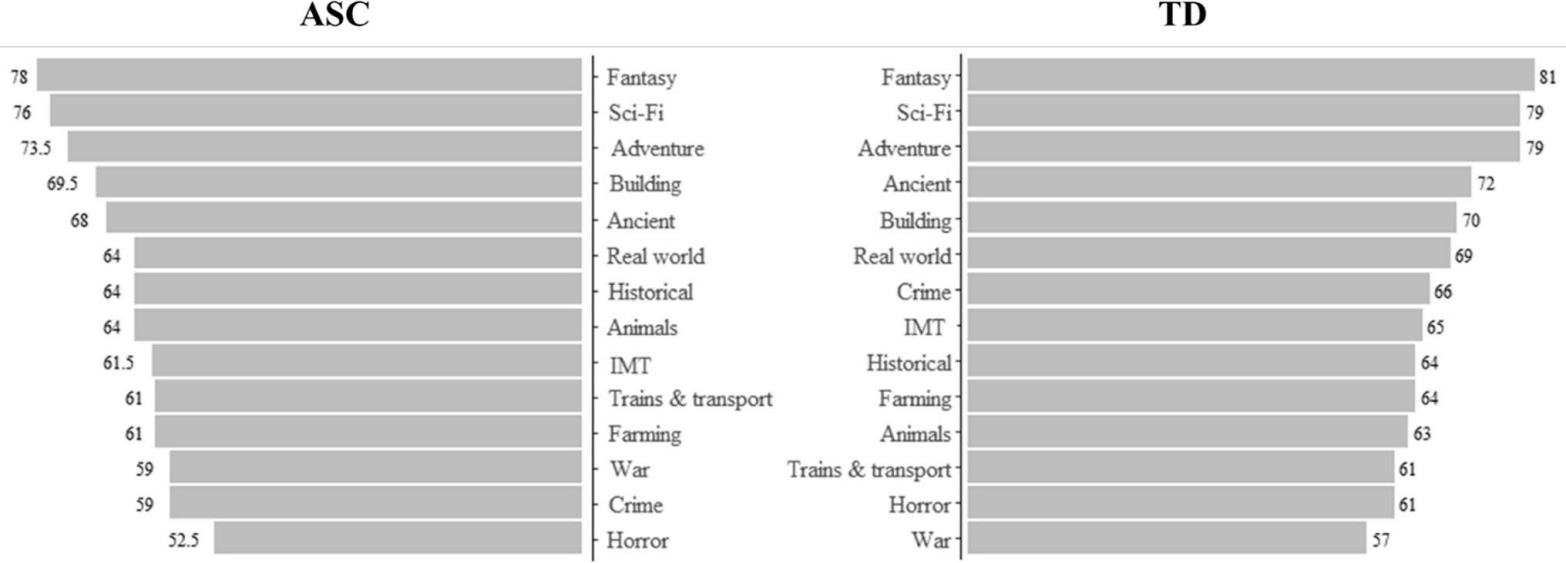
Median Ratings for Game Themes in Order for Each Group

Again, Mann–Whitney U tests (Table 5 and Fig. 2) indicated that, out of the 14 themes, those in the ASC group gave lower ratings compared to those in the non-ASC group for the adventure, ancient, real-world, crime, and horror themes. Despite this, the order of preference across themes is similar between the two groups. The only notable exception is the crime theme, which is one of the least favourite themes for the ASC group while occupying a relatively high position for the non-ASC group.

**Table 5:** Median ratings for board game themes divided by TDs and ASCs

Theme	Non ASC^	TD^	Mann–Whitney U
Fantasy	78	81	107,801.5
Sci-Fi	76	79	108,740
Adventure	73.5	79	102,139.5*
Building	69.5	70	110,580.5
Ancient	68	72	100,072.5**
Real world	64	69	102,271*
Historical	64	64	109,912
Animals	64	63	119,878.5
Industry /manufacturing /trading	61.5	65	111,094
Farming	61	64	111,200
Trains & transport	61	61	107,444
Crime	59	66	99,468**
War	59	57	116,861.5
Horror	52.5	61	104,088.5*

Mann–Whitney U Tests are Included

*p < .05; **p < .01; ***p < .001

^Minimum and maximum ratings were always 0—100

#### Game Choice and Motivation

Mann–Whitney U tests (Table 6) indicated that the ASC group gave lower ratings than the non-ASC group when indicating how important gameplay, mechanics, theme, style and components were in the choice of board games. However, the order of the ratings within each category seemed to be the same between the non-ASC and ASC groups. The only exception is that the non-ASC group were more motivated to play board games because of the emphasis on social interaction rather than strategizing. Meanwhile, this was reversed for the ASC group.

**Table 6:** TDs’ and ASCs’ rating for game choice criteria, game motivation and social aspects of board games

Category	Field	Non ASC^	TD^	Mann–Whitney U test
Game choice criteria	Gameplay	91 (17–100)	91	110,920
	Mechanics	82 (12–100)	84	114,216
	Theme	68	74	94,418.5***
	Style	68 (8–100)	72	98,751.5**
	Components	65.5	68	107,517.5
Motivations	Strategising	75 (10–100)	78	112,455
	Escapism	71	68	124,375.5
	Social interaction	70	81	83,977***
	Skill building	66.5	67	116,957.5
	Competition	61	64	104,494.5
Social importance	Gaming for social life	64	72	96,822.5***
	Importance of community	60	64	102,206.5*

Mann–Whitney U Tests are Included

*^*Minimum and maximum ratings were 0 and 100 when not otherwise specified

#### Summary of Findings

Autistic gamers showed a preference for online over in-person gaming. Also, they showed an elevated appreciation for cooperative and solo gameplay while rating party games lower than their non-ASC counterparts. The higher ratings for solo and online gaming and lower ratings for party games could be interpreted as showing that autistic people are more comfortable in their own company than neurotypicals (Baldwin & Costley, 2015). However, other findings, such as the penchant for cooperative games, show a social side to autistic players. Autistic gamers also ranked certain kinds of game mechanics more favourably than neurotypicals. These mainly included logical and systematic aspects of games, such as engine building, set collection and hand management, while ranking social elements such as storytelling, deduction and trading less favourably. This seems to mirror autistic preferences for logic, maths, and the sciences (Wei et al., 2013).

Similarly, autistic players ranked game themes revolving around transport, trains, history, and animals higher than neurotypicals, with other themes such as horror and crimes ranked lower. This overlaps with popular passions in autism (Cho et al., 2017). It also contradicts anecdotal evidence that individuals with autism are more likely to be interested in crime (Im, 2016). Similarly, autistic gamers ranked strategizing as a more important motivation for playing than socialising, which was reversed amongst neurotypicals. The results presented here help elucidate autistic individuals’ reasons for board gaming, and the dataset is made open access to aid with this.

Importantly, our results suggest that autistic people are able to find aspects of gameplay that suit their particular needs and interests. While there are differences between autistic and non-autistic board game preferences, board games have enough variety that they can accommodate a variety of preferences. That said, there are still significant areas of overlap in the preferences of autistic and non-autistic gamers, showing that there are ways to play games in mixed groups without sacrificing enjoyment. Furthermore, in line with monotropism, it appears that autistic players are playing board games for longer, and even playing individually. This suggests that board games may represent an overlap between a special interest and a preference for systemising. To further understand the reasons why autistic people may be drawn to board gaming, and the way that board gaming affects their social lives, we interviewed 13 autistic people who were board gaming enthusiasts.

## Study 2

### Methods

Thirteen autistic board game hobbyists (10 male, 3 female, age range: 24–51) from the US, Europe and the UK were recruited through board gaming social media networks and through contact information left in Study 1. All participants were avid board game players; some were also involved in their design, distribution and retail. All participants had a formal diagnosis of autism (except one who was self-diagnosed). Interviews were conducted online (via video conferencing) or in person, with participants choosing their preferred mode, and each one lasted around one hour. All participants gave full informed consent, were debriefed upon the interview’s conclusion, and paid £10. The study received full ethical approval from Edge Hill University’s ethics review board.

The semi-structured interviews focused on individuals’ experiences surrounding board games, motivations and preferences, and how they felt the hobby intersected with their condition. Example questions included ‘What do you enjoy most about the hobby?’ ‘How does gaming feature in your everyday life?’ ‘Would you say your interest in board games relates to your autistic traits, and if yes, how so?’ All interviews were recorded and then transcribed. Two independent coders then coded these transcriptions using the process outlined by Graneheim and Lundman (2004). Specifically, they each independently reviewed the data and coded each interview into subthemes. After independently coding the transcripts and creating a list of subthemes for each interview, the two coders reconvened. Together they agreed on a list of subthemes that appeared consistently across the interviews based on their independent coding. They then consensually agreed on a set of master themes that they felt best characterised the interviews and subsequent subthemes.

The method of analysis used throughout the coding process was Interpretive Phenomenological Analysis (IPA) (Eatough & Smith, 2017), a type of thematic analysis focusing on lived experience and participant voices that is particularly suited to autism research (MacLeod, 2019). IPA is beneficial for amplifying the voices of members of marginalised and minority groups, as it attempts to use the participants’ own language to form codes, themes, and subthemes (Tuffour, 2017). It is also useful when researchers are interested in moving beyond pre-conceived theory and instead want to understand how individual experiences may open new areas of inquiry (Smith et al., 2009). It is important to note the positionality of the researchers in this study. One of the researchers was unfamiliar with autism and board gaming. The other was a seasoned autism researcher and familiar with board games. This difference in backgrounds was preferable as it meant that shared observations about codes and themes were driven by the data rather than familiarity with existing research literature.

RQ1: What does hobbyist board gaming afford autistic players?

RQ2: How do they conceptualise the intersection between board gaming and autism?

## Results and Discussion

Four key themes arose from the interviews. See Table 7 for the frequency of themes within the interviews and selected quotes from participants that illustrate each theme. One coder applied the final codes. Results highlighted the benefits of board gaming for autistic individuals involving structure, friendships and escaping the outside world.

**Table 7:** Frequency count of subthemes within interview data transcripts

Themes	Frequency in Interviews	Frequency of themes
Systems are stimulating and comforting	9	38
I like the rules aspect. The first excitement with opening a new game, it’s always the rulebook for me, how it’s presented, I like to see the multiple layers to the rules. I like the structure. It’s like building with Lego. You start from ground up, see the foundation, and how to complete it. Then you build up and see the complete picture. It’s beautiful It scratches a mental itch. I love it when there’s some strategic depth, when there’s lots of options. You get to pick from many different options and many different strategies. Academic autistic people, who have high intellectual ability, including myself, find a lot of pleasure in working something out and chasing down all the inevitabilities The obvious thing that comes to mind is the structure… there are rules and you can learn all the rules. You don’t have to worry about any weird other rules that somehow everybody else knew that you didn’t get the memo about… you can safely interact in this environment without having to worry about something unexpected popping out I think autistic people really appreciate and find comfort in rigorous rules settings. I think other people with autism I’ve met, all really enjoy learning the rules for something… It’s quite anxiety inducing when I don’t understand the rules. It’s a sort of structure where there won’t be surprises that go beyond what you can expect. It’s special in that it’s sort of like a puzzle		
Passions & Escapism	9	34
I do have a thing for Sci-fi theme type games and also for beer and spirits related game. So it’s kind of like my little like themed area that I do collect a little bit I personally like the fact that it allows me to unwind and be someone else for a change… you literally become your character. Board games enabled me to get out of that headspace…to escape … just being myself, which is so pressurising, and you know it’s a break so to speak… like an island of calm The different themes…you can have a conversation with someone about it and I can talk for hours just going off different, you know, about themes within the hobby. Autistic people can be quite fixated and fanatical about their interests… with board games, there are a lot of games that do justice to the thing they are based on… it’s just a nice thing for someone who’s extremely fanatical about something to be able to get involved in it They feel comfortable chatting Pokémon, magic, Warhammer, board games, D&D. The parents see these children suddenly lighting up having these massive conversations about their world, about the stuff that they’re obsessed with, about the things that they love. They suddenly found someone that understands them We can all sit around a game and go ‘Now this is lovely. This is a great thing!’ we’re all into it. you’re allowed to be enthusiastic about this and into it with other [gamers], because they’re just as into it. Rather than being like ‘Oh, I’ve been talking for 10 to 15 min about this and no one else cares. I’ll just shut up, and you carry on doing, like, grown up stuff.’		
Games as a Social Lubricant	11	50
It’s a structured environment in which to interact with other humans. We haven’t got to worry as much about the whole insane social interaction thing…interacting via the game, via the rules of the game, that’s the centre of attention. Eyes are on the board, not on you I like the ability to socialise with other people where something else is the focus rather than necessarily each other. I mean, we obviously we talk to each other about ourselves and everything, but it’s like having that point on a face that you look at, so you make the other person think that you’re looking in their eyes, but you aren’t really, because that’s a terrifying thing to actually do. It’s having something else as a distraction I’ve got a discrete distraction to pull people back to if I feel like I don’t know what’s happening…I can’t relate or don’t know how to respond or if I should respond or if I feel like maybe there’s a stall in conversation and nobody is saying anything, you can focus on the game. There’s no sort of panicking about what’s going on socially because you can always just pull back on it whenever anything seems wrong I’m still bad at making small talk with people I don’t know. Or getting to know someone, like initially. It’s always been something I’ve struggled with. But, with gaming, you’re getting together, sitting around a table and agreeing to interact through this structured set of rules and components and things which definitely, for me has facilitated getting to know some folks… So, I think once you get to know folks, it becomes less about the structured interaction through the game and more just kind of socialising as friends around playing a game We have seen friendships forming between little groups, and gradually they come in contact with other groups, there starting to connect, and we’re starting to see bigger groups forming I used to find things that you do in board games just in video games, but I completely stopped playing video games like eight or nine years ago. It’s not because I don’t like it, but because it was taking too much time in a non social way. I probably spend almost as much time playing board games. But now it’s with people you know. it’s something I can socialise with as well		
Social Games & Deception	9	10
I like looking at psychology of people… or like copying other people’s emotions. I think it would probably surprise people that I enjoy, like interactive games, social games, party games, probably more than you might expect That would be one style of game where my autism does manifest itself … I don’t enjoy those as much, and I don’t think I’m particularly good at those. The deception, I wonder whether that’s necessarily a skill that we need to know. I mean I am an advocate of truth and something I’ve always struggled with is people lying I do love social deception games and how they are very intense emotionally. And yeah I would say part of it was that it’s a safe environment to practice like communication and deception. I don’t think I ever was really good at like. Me, I like detecting if someone was lying, at least not through like nonverbal stuff or like attitude or stuff I could get it through deduction and through like comparing like past lies and like logical inconsistencies		

Frequency of interviews refers to how many interviews possessed this theme. The theme frequency refers to the number of times these were coded within the total data. Supporting interview quotes are given below each theme in italics.

### Theme 1: Systems are Both Comforting and Stimulating

Participants discussed how board games’ intellectual challenge and strategic depth drew them into the hobby from an early age. Learning the rules of a game and figuring out how to use those rules to maximise their strategy was key to their enjoyment. The rules gave games a challenge by constraining players to specific pre-sets (time limits, hand limits, turn limits, dice rolls, and victory goals). In this way, games became like a puzzle to solve. The sense of competition, problem-solving, and accomplishment was important for participants. The ability to play games over and over and thus improve on their previous strategy or take more risks in the game was particularly rewarding.

In addition to finding the structure and repetition of the games engaging, participants also found comfort in how game play was based on a clear system. Knowing the rules meant that nothing unexpected was going to happen and it also meant everyone started the game on the same page. Each player had the same rules to follow, and they didn’t have to worry about anything unexpected happening that they might not understand. Importantly, conversations and discussions were centred on predefined, mutually understood systems. Players felt like they understood the ‘language’ of the game based on their comfort with board game systems, and so it was easy to engage other players in discussions around that game and other games. In this way, being a board game enthusiast with extensive knowledge about board games led to engaging discussions about this shared interest.

Participants reported that the rules and structure involved in playing the board game were both stimulating and comforting. Previous research by Müller et al. (2008) found that structured social environments were ideal for interactions between autistic people and others. Results of the current study support this, with all participants expressing how the rules and structured setting of board games were well matched with their autistic traits, as it gave a sense of security compared to the usual interactions where the rules are unclear (Mazurek, 2014). Board games match well with the systemising theory of autism, which explains the motivation of autistic people to rely on structure and rules to help their decision-making (Baron-Cohen, 2009).

### Theme 2: Passions and Escapism

Many people discussed how board games had become a passion. In this way, when they played board games with other gamers who were also passionate about the hobby, they could participate in what felt like meaningful conversations. In other contexts, they might feel self-conscious when talking in detail about a special interest. Through board gaming, they found people who understand why they love the things they do. This gave participants a sense of belonging and connectedness.

Inherent to this enjoyment of engaging with a special interest in board gaming, games themselves provided escapism through immersion. Participants reported feeling absorbed in a new world when playing a game, particularly when it was aligned with another passion (i.e., science fiction, fantasy, animals or history). Individuals could find many different themes and mechanics associated with games that suit their differences, preferences, needs and interests. One participant who runs a board gaming club in their community found that through playing games, their autistic attendees felt more comfortable discussing their other passions (like Pokemon, Marvel or Dungeons and Dragons) and often found that other gamers shared these passions.

Immersion not only allowed for engagement in a passion but also gave participants license to not think about real life. Participants could escape themselves by being a character and focusing on the game’s progression. More than anything, games gave them the liberty to do something purely for enjoyment and something that had no lasting ramifications as it’s ‘just a game.’ As one participant explained, *‘It enables me to just switch off my brain.’* Participants expressed that being themselves can be too pressurising but that board games are a distraction from this stress. They help manage extreme emotions by gaining comfort in the knowledge that the purpose at that moment is to have fun and not take things too seriously.

Passions and escapism allowed participants to lose themselves within their areas of interest. The wide selection of board games allowed them to choose games based on specific passions, resulting in enjoyment and satisfaction. Indeed, many common board game themes such as sci-fi, transport, animals, etc. match common passions of autistic people (Klin et al., 2007). Engaging in passions was clearly an essential component of the hobby and was helpful for reducing stress and anxiety (Attwood, 2003). Furthermore, engaging in active discussion about passions can evidently reduce autistic individuals’ difficulties in communication and social interaction (Winter-Messiers, 2007). As well as highlighting the importance of passions for adults, the current study shows the importance of passions being positive, not negative, and something that needs to be encouraged and seen as valuable, not a problem.

### Theme 3: Games as a Social Lubricant

An overriding theme in the interviews was the social side of gaming. The hobby created opportunities for making friends and joining the gaming community. One participant said, *“It’s probably my primary method of making friends”.* Participants overwhelmingly attested that games were a social lubricant, allowing them to interact socially in comforting and authentic ways.

Participants discussed how the structure of board games enhanced their ability to socialise. By being able to focus on a game, interactions were less nebulous. In this way, the game being at the root of the interaction reduced pressure and stress. Games provided common ground in conversation where there was no need to worry about small talk or trying to fit in. They already fit in with the group because of the interests they shared. “*Yeah, it’s just a medium through which to be with other people.”* Board gaming provided security to engage in meaningful conversations where the social interactions occurred in parallel with the game, which reduced pressure and led to less masking.

Players also got to know other players meaningfully by seeing how they played together. Some of their closest friendships evolved by seeing how their play styles fit with other players. Getting along in the game also made conversations outside of the game easier. It afforded an avenue to nurture friendships. Planning to play games together again, meeting up at a board gaming event, or playing games online was a way for players to socialise. Because friendships with gamers were based on shared interests, participants felt like their board game friends knew their authentic selves.

Participants also discussed how their board gaming friendship groups were a mix of autistic and non-autistic players. They felt that within these mixed groups, they were appreciated for their autistic traits, for instance, being the first to learn the rules, being the main organiser of meet-ups, or even being the most level-headed. Participants discussed how in their experience, it was prevalent for gamers to have autism or be somewhere on the spectrum, so in this way, it was not stressful to disclose their autism to fellow board gamers. Almost every participant discussed how they had moved away from playing video games precisely because they were getting so much more social enjoyment from board games, a pastime still full of strategy and replayability but one that better facilitated social interaction with real-life players.

This theme discussed how board games may work as a social lubricant. Previous research explains that while autistic individuals struggle with social skills such as communication, they still desire social interaction and friendships just like neurotypicals (Crompton et al., 2020). Coupled with research showing that board games positively affect the development of friendships (Parks & Parks, 2023) and encourage conversation and reciprocal social behaviours (Rogerson et al., 2018), the current study expands on previous research by articulating the broader motivations for gaming. Board games allow autistic individuals to find people with the same interests. They can talk about their passions, which instead of appearing tedious and creating awkward moments, is welcomed within these groups. Thanks to its straightforward rules, the game also becomes a safe place where social reciprocity can flourish.

### Theme 4: Social Games and Deception

Deception is a mechanic of some popular board games, often referred to as social deception games, that require players to hide their identities or catch which player among them is bluffing. Popular social deception board games, which follow closely from the original parlour game ‘Mafia,’ include games like Werewolf, Spyfall, Deception: Murder in Hong Kong, Battlestar Galactica and Shadows over Camelot. In these games, a randomly chosen player is dealt a character card which tells them whether they are ‘innocent’ or ‘a traitor,’ and the game’s goal is to ‘win’ as your character by either revealing the traitor or evading detection. Some of these games are relatively short and could be conceived as ‘party games.’ Others are played over several hours and have more complex game mechanics, requiring sustained attention to detect deception or deceive other players. Within the interviews, participants were explicitly asked about their enjoyment of social deception games due to research on autism and theory of mind, which suggests autistic people may struggle with bluffing and detecting deceit (Frith et al., 1994) and honesty, which suggests that autistic people dislike mistruth (Atherton et al., 2019).

Interestingly, our participants reported that, on the whole, they quite enjoyed social deception games. Some participants stated that this likely would be a surprise, as they were aware of the stereotype that autistic people would do poorly at such games. Instead, they found that the logical side of figuring out other people’s intentions was fun and something they did well. At the same time, participants reported that they often struggled when they were the traitor, as they felt it was hard to come up with a lie under pressure. That said, some participants reported that after having played social deception games for many years, they had ‘figured out’ strategies for being the traitor after observing others. Other participants felt comfortable admitting that lying under pressure was simply a skill they did not possess even after having played these games quite often. However, because lie production took place in a gamified setting and was ‘just for fun,’ they did not mind that this was a bit hard for them and still enjoyed playing these kinds of games. More than anything, the participants enjoyed how social these games were, so the shared enjoyment of the group overshadowed their unease when playing as the traitor.

Social deception games were a theme which exposed some participants’ complicated relationships with bluffing and deception. Some participants enjoyed playing them, while others did not believe they had the necessary skills or motivation. However, participants did suggest that they enjoyed the social aspects of these types of games even if they found lie production difficult. They also felt that games were a safe space to practice these skills, and they appreciated how the game allowed experimentation with these types of mechanics. This is a particularly interesting theme as there is a plethora of research that suggests not only are autistic people poor deceivers (which participants largely supported) but that autistic people have a strong preference for honesty (Atherton et al., 2019). However, this theme suggests that there are aspects of deception that autistic people enjoy and that if practised in the right setting, they are quite competent (many participants discussed observing both verbal and non-verbal cues to spot lies), and games may provide a safe space to practice these skills.

### Summary of Findings

The current study aimed to explore the lived experiences of autistic gamers to better understand why they might engage in the hobby and what benefits they associated with board gaming. Four themes emerged from the interviews, the first involved how the systems inherent to board games were both stimulating and comforting, the second discussed how board games offered escapism and overlap with passions, the third showed how games acted as a social lubricant or alternative vehicle for social communication, and finally the fourth had to do with social deception games and how these were both difficult but enjoyable.

In conclusion, these themes both support and contradict a number of influential theories of autism as understood through the lens of the board gaming hobby. First, interest in board games as explained by autistic boardgamers centres upon the structure that defines the game. This structure is inherently interesting, as it allows strategizing and improvement over time through replayability. Not only is the board gaming structure interesting, but it provides healthy boundaries within the social interactions between players. In contrast to open-ended social interactions like chit-chat at a dinner party, players are able to talk about the game and get to know people through the way they interact around the board. Importantly, these interviews contradict one of the dominant theories of autism, the social motivation theory, which suggests that autistic people are not as interested in social interactions as neurotypical people (Chevallier et al., 2012). Participants here discussed how one of the biggest draws to board gaming is the social connection they experience when playing games, including how they prefer them over less socially interactive hobbies like video games. This includes playing games that they find more difficult in order to have social experiences within groups. One can take away from these interviews the possibility that autistic people, while socially motivated, may lack the confidence to engage in unstructured social interactions (or they simply find this style of interaction less rewarding). Activities like board gaming may provide a valuable set of social constraints which allow autistic people to engage in ways that map onto their existing strengths and interests.

Study 2 interviewed autistic people who already play board games. Study 3 built on this by exploring the benefits of introducing board games to autistic individuals who were not previously involved in this hobby. To achieve this, four community centres for autistic individuals around the UK were visited. Attendees were introduced to a range of games over an afternoon play session and then focus groups were conducted to learn more about their experiences.

## Study 3

### Methods

In this mixed methods study, the researchers visited community groups for autistic adults to play a range of commercially available board games (Dixit, Codenames, Werewolf, Spyfall, Hanabi, Deception Murder in Hong Kong. For a description of what these games entail, please see boardgamegeek.com). Twenty-eight individuals took part, 16 males and 12 females aged between 18 and 60 years old. The majority of these were not regular gamers. All participants had a diagnosis of ASC and were attendees at 1 of 3 different community groups for autistic adults in the UK in Plymouth (n = 10), Maidenhead (n = 8), Huddersfield (n = 5) and a neurodiversity group at a university in Liverpool (n = 5). Four separate game sessions and four separate focus groups were conducted, one at each of the above sites. Each play session lasted for around 2 h, and participants at each site played games with each other and with the two researchers. Following the game sessions, the participants at each site were interviewed about their experiences in a focus group, which lasted approximately 45–60 min. A range of community groups were invited to participate, and all who agreed to participate were included. All individuals had a formal diagnosis of autism from a medical professional.

The semi-structured interviews focused on the game’s experiences, including preferences and challenges, and how similar board games may be used in future group sessions. All participants gave full informed consent and were debriefed upon the conclusion of the interview. The study received full ethical approval from Edge Hill University’s ethical review board.

RQ1: What could hobbyist board gaming afford new autistic players?

RQ2: How do players conceptualise the intersection between board gaming and autism?

## Results and Discussion

Two key themes arose from the interviews. See Table 8 for the frequency of themes within the interviews and selected quotes. These frequencies were based on the agreement between coders on the subthemes found across all interviews, and then each interview was recoded by one of the researchers to gain accurate frequency counts in the interviews for each subtheme and theme. Results highlighted how board gaming could be an alternative vehicle for forging social relationships and how board gaming can be both challenging but also a growing experience.

**Table 8:** Frequency count of subthemes within interview data transcripts

Themes	Frequency in Interviews	Frequency of themes
Board games as an alternative vehicle for forging social relationships	3	15
I think this is more relaxing because it gives me a chance to communicate and get to know other people like myself, whereas before, I didn’t really get along with others. I’ve had situations before where I was put under too much pressure, and I just couldn’t cope with it and I had a meltdown. The games give a distraction from that, basically. I find that this helps because when I’m playing games, I’m not reading body language and everything else If you’re playing a game with someone, or if you’re playing the game in a group, it’s not small talk, is it? It’s ‘Hey, have taken your turn yet’ or ‘hey, roll the dice,’ it’s something logical to do with the game. With games you’re not talking to someone for fun; you’re talking to them because you need to, to play the game. I feel like everyone got along a bit better than before because it felt like what we were talking about was the game. I feel like there’s less awkwardness because, by design of the game, it encouraged you to talk to each other The board games are interesting. And we were all interested. I mean, there was no one who felt left out or didn’t participate. Because when you’re on the spectrum, we find just making conversation, small talk, rather difficult. Couple that with ‘we’ve got a common interest together,’ rather than you’re trying to construct chit chat about the weather, and now you’re also interacting with other people. So it might be a strategy, or it might just be that you’re having fun playing games, but either way you’re interacting with other people It was nice to see that we’re working together to try and work things out. Because we don’t often work as a big group. I mean, we do things in small groups, but working as a whole group is really nice. Nice to see everybody because you’re maybe interacting with people that you haven’t interacted with so much in the past. And I think it’s really nice to get to know each other and see new personalities coming in Even though I didn’t like the game actually, we all had banter while we were playing and it was really nice, I really enjoyed that		
Board games can be both a challenge but also a growth experience to demonstrate and build skills	3	12
I preferred to be the characters that were you were telling the truth (at which point many members of the groups joined in to agree). Ok, we all liked to be the innocent character because you don’t have to lie. I didn’t like the aspect of lying. I think as an autistic, we can’t put on an act. We’re just ourselves all the time. So, putting on an act when we’re playing Werewolf is impossible… like other members of this group I felt uncomfortable with lying and putting on a fake persona I think part of [the game Werewolf] is the process of elimination, trying to work out who were the villagers, so to narrow down the list of possible suspects. And, what people were saying, and why were they saying it? Was there anything suspicious in their claims? If they said they got up during the night (a phase of the game), Why did they get up during the night? Because I’m on the autistic spectrum I don’t like lying and dishonesty, the old saying ‘What a tangled web we weave when first we practice to deceive.’ When we try to lie, especially when you’re not used to it, we find it quite difficult to construct a fake story and get caught out quite quickly, you tell a lie and people catch you out You learn to look closely at people, see if they look even the teeniest bit suspicious, observe what they’re saying and how they’re saying it, and try to figure out why. Body language is a real issue, I give it away, everyone knows I’m lying straight away, its blatantly obvious, body language is a big thing…. its hard as you can’t prove your lie I felt like, despite this being the most fun bit about the games, I felt like it was hard convincing other people to agree with you. That was definitely quite hard. I was a werewolf three times in a row… And to convince people that I was innocent, was quite difficult, but I did enjoy that part. Generally speaking, I sort of went for things and made multiple arguments, sort of arguing, okay, they say maybe we should consider so many arguments for all side I enjoyed the game; I was happy with the first version of the game. But the second game, when you introduce greater complexity, you introduce the robber character, I felt I was being asked to keep too many balls in the air at once. New games are more of a challenge, when I play them, I’m challenged more. So, with these games, like with my autism, I’ve been encouraged to tolerate change more I think this group has opened up a lot of confidence. I mean on my own behalf; the games have opened up a doorway		

Frequency of interviews refers to how many interviews possessed this theme. The theme frequency refers to the number of times these were coded within the total data transcripts. Supporting interview quotes are given below each theme in italics.

### Theme 1: Board Games as an Alternative Vehicle for Forging Social Relationships

Participants described how playing games acted as a vehicle for creating and maintaining friendships. Games reduced the anxiety that comes with traditional avenues for making friends. Board games, in fact, created the perfect environment for socialisation because it eliminated small talk, which participants found dull and disingenuous. Similarly, the game provided a distraction from the pressure of usual conversations, while at the same time, the game provided the topic for the talk among players. The social interaction between autistic individuals and other players, therefore, occurred naturally, without imposition. This was rewarding for the players because, while playing the game, they got to know others while avoiding awkward situations. Eventually, for some participants, the fluid interaction with others was the only reason why they enjoyed the game.

In summary, participants in these sessions expressed how playing games offered a rewarding and enjoyable alternative form of social interaction, which helped alleviate many of the social pressures they often felt in unstructured social situations. Aside from the social side of board gaming, our participants also expressed a range of other competencies that they felt could benefit from board gaming, even though these also presented significant challenges.

### Theme 2: Board Games Can Be Both a Challenge but Also a Growth Experience to Demonstrate and Build Skills

Participants described how games presented various challenges but also offered an avenue for skill development. For example, many of the deceptive/bluffing aspects of some games were problematic for some players, even though they provided a source of excitement and strategy.

Participants indicated that they did not like to lie because their non-verbal actions betrayed them.

Similarly, making up a credible lie was sometimes difficult for them. For these reasons, they felt they did a poor job in games requiring them to trick, bluff or deceive. Although individuals clearly expressed that they found deception challenging, they also noted how the games naturally help refine skills such as perspective-taking, bluffing, and reading other people. For example, participants talked at length about the way they observed other players’ behaviours to try and detect and untangle truthful vs bluffing statements in the games.

Participants also enjoyed the metacognition that these games encouraged, such as thinking about their own thinking strategies, and other players’ choices. For example, a more logical/deductive strategy was often used to detect lies. Players enjoyed challenging themselves socially by building their persuasion and debate skills. However, participants recognised that autistic individuals might find other aspects of the games challenging, particularly in relation to the complexity of the game. Despite these issues, participants discussed how games allowed them to grow their confidence.

Others noted that although they find the process challenging, this challenge allows them to learn to adapt to change.

### Summary

Autistic individuals, new to board gaming, expressed many of the same sentiments as more seasoned gamers in Study 2. They discussed the way in which board games can act as a social lubricant and the comfort found in the systems and rules inherent in games. They also discussed how they felt they struggled with the social deductive and bluffing aspects of games, yet also described the kinds of rich perspective-taking they engaged in when playing them. Unlike more seasoned players in Study 2, Study 3’s participants did not discuss game themes and passions, although this is not surprising since they were exposed to a significantly smaller range of games. Unlike Study 2’s participants, they also reported struggling more with some of the more complex rules, though this, too, is to be expected since they were less experienced.

## General Discussion

We reported on a series of studies highlighting the unique potential that board gaming may have to impact and transform the lives of adults on the spectrum. In study 1, we explored the popularity of the hobby among autistic people. As predicted, across a sample of over 1600 board gamers, we found that autism (and anxiety, conditions that often co-occur) were elevated among board gamers, while other mental health conditions were not. Furthermore, we found that the BAP was also elevated in our sample. Clinical and subclinical cut-off rates for autistic traits presented in our sample occurred at a significantly higher rate than is typically seen in the general population. This study also highlights autistic players’ preferences and motivations within this hobby. In study 2, autistic board gamers indicated that the form of structured socialisation that took place during the game suited autistic ways of being. In study 3, we introduced board games to community groups of autistic adults around the UK, finding that board gaming ‘newbies’ echoed many of the sentiments of more seasoned gamers. Games made socialising easier, and it was fun to problem-solve within a set of rules. Perhaps most importantly, study 3 showed how board games could bring together diverse groups of autistic people who often have different needs and interests. Groups stated how they had come together for the first time rather than interacting within their smaller, well-established friendship circles. Together, our results suggest that board games may occupy an essential place in the social lives of autistic people. It also indicates that this may be a valuable hobby for autistic people, as it may benefit them cognitively and socially in several ways.

Improving mental health outcomes for autistic people is a pressing matter for autism research (Crane et al., 2019). Research suggests that as autistic people age, they are less likely to experience gains in quality of life compared to neurotypical people. This disparity is particularly pronounced for autistic people diagnosed later in life, which is a growing proportion of the autistic population (Atherton et al., 2021). To increase the quality of life for autistic people, understanding and promoting healthy leisure patterns may be essential (Potvin et al., 2013). A plethora of research suggests that friendship and social connection protect mental health (for a review, see King et al., 2016), with shared interests as a key factor in establishing relationships (Yang et al., 2011). This may be particularly important for autistic people who exhibit passions, which can be a source of bonding with others which can lead to an acceptance of atypical behaviours (Sosnowy et al., 2018), and allow for a more immediate connection and purpose within a social group (Chan et al., 2022).

Our findings suggest that board games may be particularly beneficial for autistic adults by allowing them to interact socially in a way that is suited to their social style. Research shows that autistic people struggle to socialise in more open-ended or loosely structured settings that require small talk (Pfeiffer et al., 2017). In situations where there is no structure to conversation, autistic people can miss social cues leading to social anxiety (Livingston et al., 2019), resulting in avoiding social situations where they might face rejection (Hull et al., 2017). This mismatch between neurotypical social styles and autistic ways of being may be at the heart of the many studies finding that autistic people experience significantly more loneliness than neurotypicals (Umagami et al., 2022). Despite a need for social connection, autistic people may feel that their social skills preclude them from entering social situations where they can cultivate friendships (Stice & Lavner, 2019).

Board gaming offers a unique solution to these issues by removing the small talk and moving the attention from other implicit social cues such as body language, which autistic people often find challenging, to the game and its rules. Additionally, players can use a common language about the game (Knight et al., 2019), allowing them to discuss their passions with others who share these interests. Finally, the structure of gaming allows for further interactions over time, as indicated by participants who were both seasoned gamers (study 2) and new to gaming (study 3). Because board games can be played at a slower pace than video games or sports, players can converse more freely during the game. Over time, they can have the unstructured interactions that autistic people often find difficult to have immediately or with strangers.

Similarly, board gaming offers autistic people self-efficacy as it depends on cognitive skills particularly adapted to autistic ways of being. Since the earliest conceptualisations of autism, the condition was characterised as one where individuals enjoyed understanding systems and rules (Kanner, 1943). After more investigation, researchers found that autistic people (Wheelwright & Baron-Cohen, 2001) and their family members (Baron-Cohen, 1998) were more likely to be involved in the STEM fields, leading to the influential ‘empathising-systemising’ theory of autism (Baron-Cohen et al., 2001). In this theory, autistic people are posited to be ‘hyper-systemisers,’ meaning they are inclined to figure out the rules or structure underlying incoming information (Baron-Cohen, 2006). While the assertion that autistic people are not as empathetic as other people (a position taken by the systemising theory) has been the source of much debate (Duffy & Dorner, 2011), there is evidence that an autistic strength is the ability to decode the underlying ‘systems’ at play in our world (Greenberg et al., 2018). Systemising may also lie at the heart of autistic passions, as discussed, for instance, in interviews with highly successful autistic people (South & Sunderland, 2022).

Given autistic people’s penchant for systemising, the focus that board games place on understanding rules and the social aspects of conversing about the underlying structure of games makes this a particularly valuable pastime for autistic adults. Research shows that autistic people do particularly well in occupations where they can work with structures and passions (Bross & Travers, 2017). Few studies have focused on the potential for hobbies that build on an understanding and enjoyment of systems and passions. While several studies show that autistic people enjoy video games, research suggests that video games can become problematic for autistic people who, possibly in response to developing a special interest in video games, are more likely to meet the criteria for video game addiction (Coutelle et al., 2022). As discussed by participants in Study 2, board games offer similar pleasures to video games, while being more social. While they can be enjoyed by themselves online, all games can be played with others, and some games can only be played with others. The sociality of board games seemed to be particularly important for participants. In this way, board gaming may provide a vital opportunity for social networking for autistic people. This finding is also echoed in research on tabletop role playing games with autistic players (Atherton, Hathaway, et al., in press), where results showed that role playing through a character allowed for a deep bond with fellow players in a way that felt particularly natural and authentic.

Future research may want to understand how board gaming as a hobby can be used to benefit the lives of autistic people; research may also wish to focus on sub-populations with restricted language and the use of language-based games in relevant skill-building. Research comparing mental health outcomes in autistic board gamers and video gamers may be useful, as our research suggests that board games may offer advantages to video games. There is also scope to investigate board gaming interventions for autistic children and adults. Our research indicates that social and cognitive skills are helped through board gameplay, which may be particularly beneficial to autistic people. Investigating the benefits of board gaming in a controlled study would be an essential contribution.

There are several limitations to this study that would benefit from further research. First, this study focused on individuals with the cognitive capacity to play modern board games, which may exclude some individuals on this autism spectrum. That said, given that many commercial board games have now produced child-friendly versions of games, it may be that with the right support individuals with high cognitive needs may still be able to engage in the hobby with support and simpler gaming formats. One recent study has investigated board gaming in an adult autistic population with co-occurring intellectual disabilities (Atherton et al., 2024) and found similar benefits in this sample, suggesting that board games may be a useful hobby across the spectrum.

Another limitation of this study is that while approximately 1/3 of the sample was comprised of people with non-white ethnic backgrounds, the majority of participants were White, male, and highly educated compared to the global population. This homogeny is also found among board gaming hobbyists, including board game designers, who are disproportionately white males (see Dias, 2023, for a review). Given that autism is also disproportionately diagnosed in males over females, and in White over minority children (Shenouda et al., 2023), it may be that this sample again speaks to the fact that a specific population may be more likely to both receive an autism diagnosis and be introduced to board gaming. Future research should focus on recruiting more diverse samples and exploring how board games may be beneficial to the wider autistic population, including females and those with minority ethnic identities.
